# Under-5 Malaria and Fever Morbidities as Correlates of Anaemia in Niger: A Heteroscedasticity-Consistent Ordered Probit Approach

**DOI:** 10.3390/ijerph21121687

**Published:** 2024-12-18

**Authors:** Thonaeng Charity Molelekoa, Abayomi Samuel Oyekale

**Affiliations:** Department of Agricultural Economics and Extension, North-West University Mafikeng Campus, Mmabatho 2735, South Africa; abayomi.oyekale@nwu.ac.za

**Keywords:** anaemia, malaria, febrile infections, under-5 children, Niger

## Abstract

**Background**: The relationship between malaria/other febrile infections and anaemia among under-5 children is a subject of significant policy relevance among African health policy makers. The international significance of addressing anaemia is prominently underscored in the third Sustainable Development Goal (SDG). This paper therefore analysed the effect of malaria/other febrile infections and other maternal and child’s demographic variables on the prevalence of anaemia in Niger. **Methods**: We utilized the under-5 children’s module of the Malaria Indicator Survey (MIS) for 2021, which was collected from women of reproductive age (15–49) in selected households. The data were analysed with heteroscedasticity-consistent ordered probit regression model. **Results:** The results showed that 73.73% of the children was anaemic, while malaria and other febrile infections were present in 14.00% and 33.87%, respectively. Anaemia was highest in the Tillaberi and Dosso regions, where 84.12% and 79.12% of the children were anaemic. The ordered probit regression revealed that anaemia was promoted by malaria, other febrile infections, being a male child, second of multiple birth, and birth order, while wealth index, age, urban residence, and access to newspaper and television reduced it. **Conclusions:** Anaemia remains a major public health problem among under-5 children in Niger. A comprehensive healthcare intervention to address the problem should consider regional, sectoral, and gender differences in the incidences, with drastic efforts towards prevention of malaria and other fever-inducing illnesses. In addition, interventions to promote households’ economic status, reduce maternal fertility, and facilitate preventive practices through nutrition enhancement and health-related media programs hold some promise.

## 1. Introduction

Anaemia and fever are health problems of significant public health concern, particularly among under-5 children in developing countries [1]. Children under the age of five are said to be anaemic when they have a low concentration of blood haemoglobin below 11 g/dL [2,3]. Moreover, fever in children is diagnosed at the body temperature of 38.5 °C or more (101.3 °F) for children older than three months and 38.0 °C (100.4 °F) or more for those who are three months or less [4]. Some statistics have shown that around 43% of under-5 children are anaemic globally, while fever is the leading cause of hospital admissions among these children [5,6]. In sub-Saharan Africa (SSA), 28.5% of under-5 children are anaemic [7,8]. The adverse health consequences of anaemia in children include stunting, the impairment of cognitive development, immune system compromise, and a high risk of morbidity and mortality [9].

In Niger, different interrelated health issues such as infections and malaria manifest as severe fever that impact under-5 children [10,11,12,13]. Therefore, fever often indicates an underlying infection, although diagnosing the exact cause can be challenging, especially in developing countries where malaria is commonly presumed [14]. By age five, children in these countries experience around 40 fever episodes, often managed with antibiotics or antimalarial drugs [15]. Specifically, fever-related illnesses and anaemia are remarkably interlinked and contribute to high rates of severe morbidity and mortality among under-5 children [16]. This emphasizes the need for thorough research and targeted interventions to promote Nigerien children’s health, for the reduction of child mortality and promotion of cognitive and physical development [17].

Furthermore, anaemia results from insufficient red blood cells or haemoglobin levels, which are crucial for transporting oxygen within the body’s cells [18,19]. Anaemia promotes a reduction in oxygen delivery to the body’s cells, causing symptoms like fatigue and shortness of breath [17]. The causes of anaemia in under-5 children include poor diet, infections, inflammation, chronic diseases, and genetic disorders [20,21]. However, iron deficiency is a primary cause of anaemia, along with deficiencies in folate, vitamins B_12_, and Vitamin A [21]. Nigerien children face significant nutritional challenges due to growing poverty and inadequate access to nutritious food [22]. Moreover, 80% of the population living in rural areas has inadequate access to essential services like clean water, healthcare services, and education [22]. The cumulative impacts of these deprivations influence a child’s health with serious survival consequences.

Several studies have shown the socioeconomic factors influencing anaemia severity among under-5 children. Specifically, it had been noted that the effect of anaemia and other febrile infections can manifest with severe symptoms, including convulsions [8,23]. In some empirical analyses, fever morbidity was found to increase the probability of being anaemic [24]. Also, Elmardi et al. [25] found anaemia prevalence to be higher among children with malaria or a two-week history of fever. Some other authors with similar findings include White [26], Kweku et al. [27], and Stoltzfus et al. [28]. However, other factors such as twin birth [29,30,31], small size at birth, higher birth order, recent fever, and distance to healthcare are linked to higher anaemia levels [30] and altitude [32,33].

Understanding fever and anaemia’s relationship is crucial for achieving Sustainable Development Goal 3. Given the complex relationship between fever and anaemia in young children, it is essential to conduct thorough studies to inform evidence-based interventions and policies, particularly focusing on the socioeconomic factors that contribute to these health challenges in Niger [33]. This study therefore provides some valuable insights into the effect of malaria/other febrile infections and other maternal demographic variables on anaemia prevalence among under-5 children in Niger, utilizing data from the Demographic Health Survey (DHS). This study seeks to identify some key correlates of anaemia infection among under-5 children in Niger and propose some intervention strategies.

## 2. Materials and Methods

### 2.1. The Data

We used the Niger’s Malaria Indicator Survey (MIS) for 2021 in this study. The survey was conducted through the DHS programme by the l’Institut National de la Statistique (INS) from 12 August to 27 October 2021 through financial and technical assistance from by the United States Agency for International Development (USAID), the government of the Republic of Niger, the United States President’s Malaria Initiative (PMI), Catholic Relief Services (CRS), and the National Malaria Control Program.

**Figure 1:**
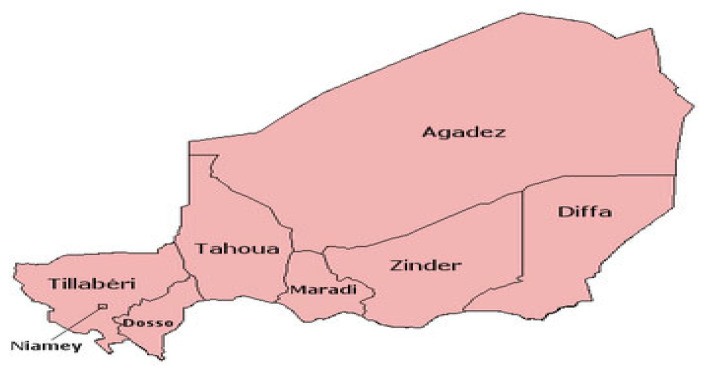
Map of Niger showing the different regions. Source: https://en.wikipedia.org/wiki/Regions_of_Niger (accessed on 20 October 2024).

The sampling targeted every eligible household in Niger, with adequate urban/rural and regional representativeness (see Figure 1 for the list of regions). Four survey instruments were used for data collection, which were household, women’s, biomarker, and field survey outcome questionnaires. This paper used the data collected with the women’s questionnaire, focusing on those who were 15–49 years of age and eligible children who were 6–59 months old. Mothers who consented to anaemia and malaria tests had their children’s blood collected for haemoglobin and rapid malaria tests, respectively. In addition, children’s temperatures were measured with thermometers.

The data collection protocols were pre-tested by 12 enumerators and 8 health officers who went through some collective and specialized trainings for two weeks. Between 12 July and 7 August 2021, the general enumerators, comprising 50 qualified persons (16 men and 34 women), were trained on the procedures and ethical requirements for the survey. The field surveys were conducted among 210 cluster samples. Due to insecurity, interviews were not conducted in two Tillabéri clusters and one cluster from Diffa. Therefore, the survey took place in a total of 207 clusters.

At the end of the survey, the number of selected households was 1272 and 3694 in urban and rural areas, respectively, while 1183 and 3546 were successfully interviewed. The number of eligible women (15–49 years of age) in the selected households was 1690 and 4263 in urban and rural areas, respectively, while those who granted their consent to participate and completed the surveys numbered 1619 and 4176 [34].

### 2.2. Limitations of the Study

One of the major limitations of this study is that not all the eligible women eventually participated. Although the response rate was quite high, the omission of some important groups could have biased our estimators. In addition, among the eligible children, many of them objected to taking their blood samples for the haemoglobin test and rapid malaria tests. However, since our sample for this study was primarily based on the state of anaemia, some level of bias could have been introduced by the non-participants.

### 2.3. Ethical Approval and Interview Consent

The protocols for conducting the survey as well as the tests for haemoglobin level and parasitaemia were reviewed and approved by the National Ethics Committee for Health Research (CNERS) of the Ministry of Public Health of Population and Social Affairs and the ICF Institutional Review Board. Also, the parents or caregivers of the children who participated in the biomarker testing were informed of the risks and benefits of participation to secure their final authorization. The results of the tests were communicated to the parents/caregivers, while some severe cases were referred to the nearest healthcare centres.

### 2.4. Estimated Econometric Model

We used heteroscedasticity-consistent ordered probit regression to analyse the data with the variance of error modelled by the households’ wealth index. The ordered probit model is appropriate when the dependent variable is more than two groups and they are ordinally coded. In the proposed model, there are four ordinal groups of anaemia, coded as 1 for severe, 2 for moderate, 3 for mild, and 4 for non-anaemic. It should be noted that the values of each group are irrelevant, only a particular order of arrangement should be followed. The model estimates a linear relationship of some independent variables and some unknown cutpoints. The estimated probability of observing an outcome $y_{j}=h$, where h=1,2,3,4…H is equivalent to the likelihood that the sum of the linear function and the error term is in the range of the cutpoints that are associated with the outcome.

(1) $$\mathrm{Pr}\left(y_{j}=h\right)=Pr(k_{h}<X_{j}\beta +e_{j}\leq k_{h+1})$$

(2) $$\mathrm{Pr}\left(y_{j}=h\right)=\Phi \left(k_{h+1}-X_{j}\beta \right)-\Phi \left(k_{h}-X_{j}\beta \right)$$

$y_{j}$ refers to the outcome variables, $X_{j}$ are the explanatory variables which are malaria infection (yes = 1, 0 otherwise), other febrile infections (yes = 1, 0 otherwise), mother’s age (years), Diffa region (yes = 1, 0 otherwise), Dosso region (yes = 1, 0 otherwise), Maradi region (yes = 1, 0 otherwise), Tahoua region (yes = 1, 0 otherwise), Tillaberi region (yes = 1, 0 otherwise), Zinder region (yes = 1, 0 otherwise), Niamey (yes = 1, 0 otherwise), urban (yes = 1, 0 otherwise), altitude (meters), primary (yes = 1, 0 otherwise), secondary (yes = 1, 0 otherwise), tertiary (yes = 1, 0 otherwise), household size, male headship (yes = 1, 0 otherwise), head age (years), read newspaper (yes = 1, 0 otherwise), listen to radio (yes = 1, 0 otherwise), watch television (yes = 1, 0 otherwise), 2nd birth order (yes = 1, 0 otherwise), 3rd birth order (yes = 1, 0 otherwise), 4th birth order (yes = 1, 0 otherwise), 5th birth order (yes = 1, 0 otherwise), 1st of multiple (yes = 1, 0 otherwise), 2nd of multiple (yes = 1, 0 otherwise), gender (male = 1, 0 otherwise), and child age (months). $k_{h}$ is the cutpoints, and $\Phi \left(.\right)$ is the cumulative distribution function. The conventional ordinal probit assumes that the error is independently identically distributed (iid) normal with constant variance. When this assumption is violated, the estimated parameters are going to violate the BLUE (best linear unbiased estimator) assumptions. This will compel the estimation of a heteroscedasticity-consistent model where the variance of the error can be modelled as a multiplicative or additive function of selected independent variables [35]). In STATA 18, which was used for data analysis, the hetoprobit command estimated the log of standard deviation as a linear function of explanatory variables. In this study, the estimated model is presented as follows:

(3) $$ln\sigma_{j}=z_{j}\pi$$

where $z_{j}$ is the household’s wealth index. When the error variance is different across different groups, Equation (2) becomes the following:

(4) $$\mathrm{Pr}\left(y_{j}=h\right)=\Phi \left(\frac{k_{h+1}-X_{j}\beta }{\exp\left(z_{j}\pi \right)}\right)-\Phi \left(\frac{k_{h}-X_{j}\beta }{\exp\left(z_{j}\pi \right)}\right)\;$$

To ensure the identifiability of the estimated models, Equations (2) and (3) are without any constant term. The analysis was also implemented with due recognition of the likelihood of multicollinearity among the variables. Therefore, the variance inflation factor (VIF) was used to evaluate the presence of multicollinearity.

## 3. Results

### 3.1. Descriptive Statistics of Selected Mothers’ and Children’s Demographic Variables

Table 1 presents the descriptive statistics of selected demographic variables across mothers and children in Niger. The table shows that the number of observations is 4142. The results revealed that the average age of the mothers was 28.88 years. The Maradi region had the highest number of children with 25.46%. This was followed by the Zinder, Tahoua, and Tillaberi regions with 23.14%, 20.21%, and 13.42%, respectively. It was also revealed that 16.23% of the children resided in urban areas. The average altitude of the regions was 354.43 m. In terms of education level, 75.34% of the mothers had no form of formal education, while primary education was attained by 14.98%. The average household size was 8.46, and the average age of the household heads was 42.53 years. In terms of media/sources of information used by households, 25.69% were listening to radio, compared to 12.79% who were watching TV and 1.51% who were reading newspapers. Furthermore, 96.99% of the children were singletons. Based on the birth order, 59.28% were first-born and 35.37% were second-born. The results further revealed that 50.86% of the children were male and the average age was 31.36 months. The average wealth index was 2579.24.

### 3.2. Malaria and Fever Morbidity Among Under-5 Children

Table 2 shows the distribution of malaria and other febrile infections across the regions and economic sectors in Niger. It reveals that 14.00% and 33.87% of the children had malaria and other febrile infections, respectively. Among those with malaria, the majority were from the Zinder region (23.10%) and Dosso region (20.69%). Moreover, among those children with other febrile infections, the majority were from Zinder (20.67%) and Tillaberi (17.89%). Across the economic sectors, rural children accounted for 83.28% and 84.39% of those with malaria and other febrile infections, respectively.

### 3.3. Distribution of Anaemia Across Selected Demographic Variables

**Figure 2:**
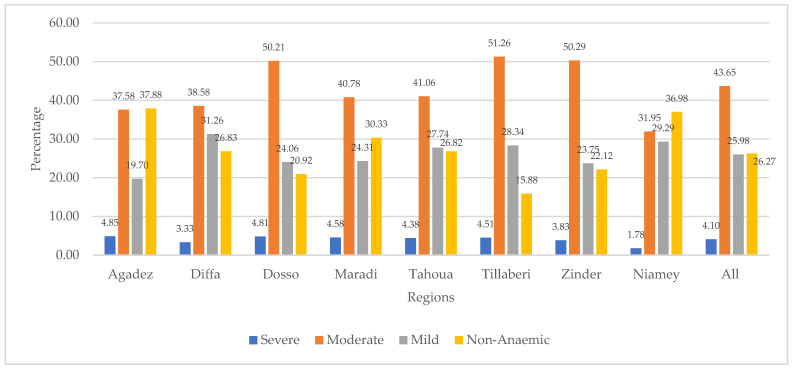
Distribution of children’s anaemia levels across regions in Niger.

Figure 2 presents the distribution of children’s anaemia levels across the regions. The figure shows that 4.85% of the children from the Agadez region were severely anaemic. Other regions with relatively high severe anaemia were Dosso (4.81%), Maradi (4.58%), and Tillaberi (4.51%). The figure also shows that majority of children in Niger were moderately anaemic, with those from Tillaberi (51.26%), Zinder (50.29%), and Dosso (50.21%) being among the highest. Mild anaemia was highest in Diffa (31.26%), Niamey (29.29%), Tillaberi (28.34%), Tahoua (27.74%), and Maradi (24.31%). The results further show that Agadez had the highest (37.88%) number of children who were not diagnosed with anaemia. This was followed by the Niamey (36.98%), Maradi (30.33%), and Diffa (26.83%) regions.

**Figure 3:**
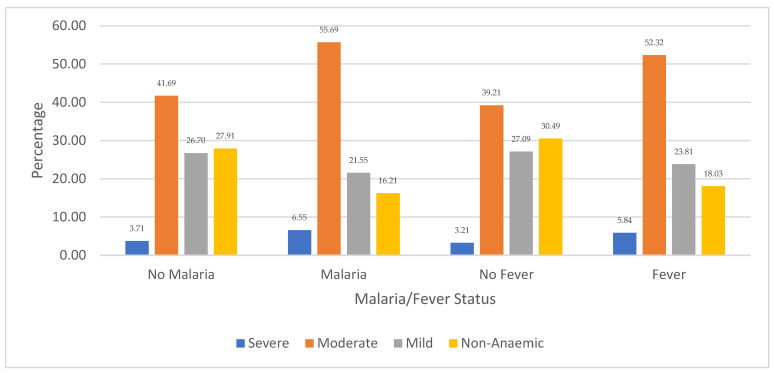
Distribution of children’s anaemia levels across malaria infection and other febrile infections in Niger.

Figure 3 shows the distribution of children’s anaemia levels regarding malaria and fever comorbidity in Niger. The results revealed that 6.55% of the children in Niger who were diagnosed with severe anaemia were infected with malaria, while 5.84% had other febrile infections. Among the children who had moderate anaemia, the majority (55.69%) were infected with malaria while 55.32% experienced fever episodes. Also, 41.69% of moderately anaemic children were not infected with malaria, and 39.21% did not report fever. Regarding children with mild anaemia, 27.09% had no fever and 26.70% had no malaria. It is also revealed that 30.49% of Nigerien children who were non-anaemic did not experience any fever episodes, while 27.91% were not infected with malaria. The figure also shows that non-anaemic children with other febrile and malaria infections were among the least, with 18.03% and 18.21%, respectively.

**Figure 4:**
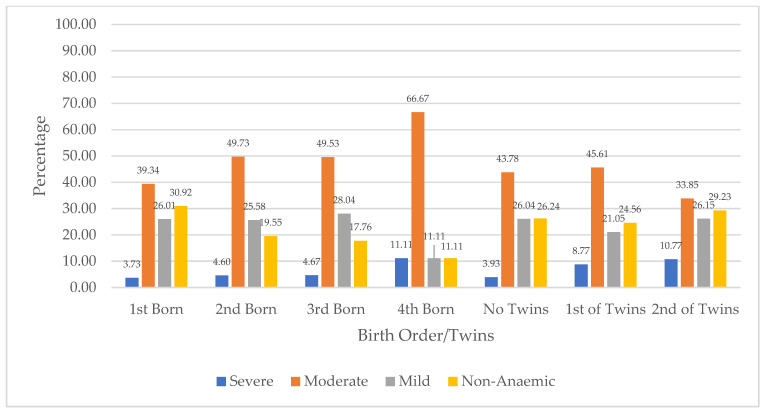
Distribution of children’s anaemia levels across birth order and twins in Niger.

Figure 4 presents the distribution of children’s anaemia levels based on their birth orders and multiple birth in Niger. The figure shows that among children with severe anaemia, with regard to birth order, 11.11% were the fourth-born in the family, compared to 3.73% who were the first-born. The figure further shows that across those who were twins, severe anaemia was higher among second children with 10.77% compared to 8.77% for first children. In terms of children with moderate anaemia across birth orders, 66.67% were the fourth-born, compared to 31.34% who were the first-born. Moderate anaemia was higher among children who were the first child of twins (45.61%), compared to the second-born of twins (33.85%). Mild anaemia across birth orders was highest among the third-born (28.04%) compared to 26.01% for the first-born. Among twins, mild anaemia was higher among the second-born (26.15%) compared to the first-born (21.05%). Also, 30.92% of children who were first-born were not anaemic, compared to those who were fourth-born (11.11%). In addition, the absence of anaemia was higher among the second-born of twins (29.23%).

### 3.4. Determinants of Anaemia Among Under-5 Children

Table 3 presents the results of the ordered probit regression. The included variables were examined for multicollinearity using the variance inflation factor (VIF). The computed value of 2.39 shows that multicollinearity was not a serious problem [36]. Also, the model was examined for heteroscedasticity with the variance of error being modelled by the wealth index. The results showed that the lnsigma parameter that was generated with the wealth index showed statistical significance (*p* < 0.05). Therefore, heteroscedasticity was a problem, and modelling the variance of error by the wealth index was appropriate. The model was run with the inclusion of the setting of the survey’s sampling design. Therefore, the survey setting menu of STATA 17 software was used for data analysis. The results revealed that the model produced a good fit to the data, with computed F-statistics being statistically significant (*p* < 0.01).

The ordered probit regression results in Table 3 show that malaria infection significantly reduced the probability of being non-anaemic (*p* < 0.05). The marginal parameters revealed that infection with malaria increased the probability of severe and moderate anaemia by 1.10% and 4.36%, respectively, while it reduced the probability of mild anaemia by 1.34% and non-anaemia by 4.11%. Similarly, the parameter of other febrile infections is with a negative sign and statistically significant (*p* < 0.01). This implies that other febrile infections reduced the probability of being non-anaemic. Marginally, infection with other febrile infections significantly increased (*p* < 0.01) the probability of being severely and moderately anaemic by 1.88% and 7.67%, respectively, while it reduced the probability of mild anaemia by 2.26% and being non-anaemic by 7.29%.

Although none of the regional parameters showed statistical significance (*p* > 0.10), the sectoral parameter of the ordered probit (urban residence) is statistically significant (*p* < 0.01) with a positive sign. This implies that urban children had a higher probability of being non-anaemic. The marginal parameters showed that urban residence reduced the probability of being severely anaemic and moderately anaemic by 2.04 and 11.30%, respectively. However, residence in urban areas increased the probability of being mild anaemic and non-anaemic by 2.00% and 11.34%, respectively.

Among the variables that captured access to media, the parameters of reading newspapers and watching television showed statistical significance (*p* < 0.05). These results indicate that children whose mothers read newspapers had a higher probability of being non-anaemic. Based on the marginal parameters, reading newspapers by under-5 children’s mothers reduced the probability of being severely and moderately anaemic by 1.93% and 12.11%, respectively, while it increased the probability of mild anaemia by 1.56% and non-anaemia by 12.48%. Furthermore, children whose mothers watched television had a significantly higher (*p* < 0.05) probability of being non-anaemic. The marginal parameters further revealed that access to television by the mothers of under-5 children reduced the probability of being severely and moderately anaemic by 0.98% and 4.87%, respectively, while it increased the probability of mild anaemia by 1.10% and not being anaemic by 4.76%.

Among the variables that were included to capture birth order, the parameters of second birth order and fifth birth order showed statistical significance (*p* < 0.05). These results revealed that being second-born reduced the probability of being non-anaemic. Furthermore, being second-born increased the probability of severe and moderate anaemia by 0.91% and 3.84%, respectively, while it reduced the probability of mild anaemia by 1.09% and being non-anaemic by 3.66%. Moreover, being fifth-born increased the probability of being non-anaemic, while it decreased the probability of severe, moderate, and mild anaemia by 3.32%, 4.55%, and 27.24%, respectively. In addition, being fifth-born increased the probability of being non-anaemic by 76.06%.

Among the variables that captured multiple birth, the parameter of being the second-born of a multiple birth showed statistical significance (*p* < 0.05) in the general ordered probit regression, as well as marginal parameters for moderate anaemia and non-anaemia. The results revealed that being the second-born of a multiple birth reduced the probability of being non-anaemic. Also, being the second-born in a multiple birth increased the probability of moderate anaemia by 13.04%, while it reduced the probability of non-anaemia by 12.12%.

The parameter of the gender of the child is statistically significant (*p* < 0.05) in the ordered probit model, as well as marginal parameters of moderate anaemia and non-anaemia. The results indicate that male children had a lower probability of being non-anaemic. Also, male children had their probability of being moderately anaemic increased by 3.02%, while their probability of being non-anaemic decreased by 2.89%. The parameter of children’s age is statistically significant (*p* < 0.01) across all the results. These showed that a unit increase in a child’s age increased the probability of being non-anaemic. The marginal parameters revealed that a unit increase in a child’s age reduced the probability of severe and moderate anaemia by 0.09% and 0.40%, respectively, while it increased the probability of mild anaemia and non-anaemia by 0.11% and 0.38%, respectively. Finally, the parameter of the household’s wealth index showed statistical significance (*p* < 0.05). The results showed that if the wealth index increases by a unit, the probability of being non-anaemic reduces. Specifically, among the estimated marginal parameters, the wealth index increased the probability of moderate and mild anaemia, while it reduced the probability of severe anaemia and being non-anaemic.

## 4. Discussion

Malaria infection is one of the major determinants of under-5 anaemia. More importantly, Africa remains the hotspot of malaria. Some statistics have shown that in 2022, 95% of the 236 million global malaria cases was in Africa, where most of the recorded deaths also occurred [37]. Similarly, SSA accounts for about 88.00% of global malaria mortalities [38], while under-5 children account for 80% of malaria mortality in Africa [39]. Our findings have shown that malaria incidence among the selected under-5 children was 14.00%. The implication is that Niger’s under-5 children have a relatively high burden of malaria, and existing interventions to address malaria like the use of insecticide-treated nets are either being jettisoned or ineffective. The fundamental problems with malaria eradication in Niger can be viewed considering households’ poverty, the poor coverage of healthcare facilities, the development of resistance to medication by malaria pathogens, and a low level of education [40,41,42].

It should be noted that emphasizing malaria prevention among under-5 children is of significance in addressing child mortality. In some previous studies, the prevalence of malaria among under-5 children varied between 0.7% [43] and 80.3% [44]. Although some studies reported less than 10% positive malaria cases [45,46,47,48,49], Ilol et al. [44] and Tusting et al. [50] reported 79.8% and 80.3%, respectively.

The results also showed that the incidence of other febrile infections was 33.87% among the selected under-5 children. Fever is one of the most common symptoms of major illnesses among under-5 children, including measles, malaria, diarrhoea, and other bacterial and viral infections [51,52]. In some previous studies, incidences of fever were 29.60% in Zambia [53] and 33.10% in Bangladesh [54]. A study by Crump et al. [55] reported that although 60.7% of under-5 children in Tanzania had malaria, it was only in 1.6% that malaria had caused fever. In another study, D’acremont et al. [14] found that among children with fever, viral infections were diagnosed in 70.5%, bacterial infections in 22.0%, and parasitic infections in 10.9%.

The findings also revealed a high incidence of anaemia among Nigerien under-5 children. With only 26.27% of the children being non-anaemic and 4.10% severe cases detected, anaemia among under-5 Nigerien children is a problem of serious public health concern. It should be noted that global under-5 anaemia prevalence has been estimated at 16.42% [56], while that for SSA is 59.9% [32]. Seifu and Tesema [30] also estimated that 76.60% of 6–23-month-old children in SSA were anaemic. In some nationally representative studies, anaemia prevalence rates were 49.40% in Sudan [25], 61.30% in South Africa [57], 47.5% in Sudan [58], 65.9% in Ghana [59], 51.8% in 6–36-month-old Swaziland children [60], 71.93% in 6–23-month-old Ethiopian children [61], 70.9% in Togo [62], 53.22% in Uganda [63], and 58.6% in Tanzania [64]. Other studies that were not nationally representative found under-5 anaemia prevalence rates at 11.10% in Nigeria [65], 3.9% in the United States of America [66], and 51.3% in rural central and western China [67].

Our findings showed that malaria and other febrile infections promoted under-5 anaemia. Malaria and anaemia are interlinked because at the initial stage when malaria parasites infect the blood, there is massive internalization and degrading of haemoglobin from the red blood cell [68]. The finding in this study is in line with some previous studies that reported a positive association between malaria and anaemia among under-5 children [69,70,71]. Our finding is, however, contrary to those of Elmardi et al. [25] among children under 24 months in Sudan. Other febrile infections also promoted anaemia among under-5 children. The channels through which fever influenced anaemia can be viewed from the fact that it is one of the major symptoms of malaria, and febrile infection promotes the destruction of red blood cells. In addition, some illnesses with fever symptoms are closely linked to infections and inflammation that decrease red blood cell production [72]. This finding is in line with the findings of Ewusie et al. [73], Semba et al. [24], Zhao et al. [74], and Gebrehaweria and Lemma [75]. However, the finding is contrary to that of Elmardi et al. [25].

Contrary to the findings of Gebrehaweria and Lemma [75], regional variables did not significantly influence anaemia among under-5 children. Although the parameters of the regional variables are statistically insignificant, some differences were obtained in the levels of anaemia across the regions. Specifically, in comparison to other regions, the anaemia incidence among children was lowest in the Agadez and Niamey regions. It should be noted that Niamey is the capital of Niger, with the highest population density, while Agadez has the lowest population density. In terms of anaemia among women of reproductive age, UNICEF [22] noted that anaemia was highest in the southwestern part of Niger, where inadequate access to clean drinking water was also predominant.

The sector of residence was found to significantly influence anaemia among under-5 children. Specifically, urban children had a lower probability of being anaemic. This is expected because of prevailing social services, such as healthcare facilities in urban centres, and the likelihood of lower income and multidimensional poverty in urban households. It should also be noted that the lower prevalence of anaemia in urban areas can be due to the expected higher literacy of urban mothers [76]. Our finding is in line with those of Khan et al. [77] and Dey et al. [76].

The wealth index was found to promote the non-anaemia condition among under-5 children. The level of wealth of under-5 children’s parents will influence the standard of living through materials for housing, asset acquisition, and the affordability of nutritious food. Our finding is in line with the findings of Dey et al. [76], who found a higher likelihood of anaemia among children from low and medium living standard. The finding also corroborates those of Assunção et al. [78] in Brazil and Mamiro et al. [79] in Tanzania. Gebrehaweria and Lemma [75] also found that community poverty promoted anaemia among under-5 children in Ethiopia, while Zhao et al. [74] and Xin et al. [80] found a higher prevalence of anaemia among children from low-income households. Relatedly, access to newspaper and television reduced the likelihood of anaemia among under-5 children. Specifically, newspapers and televisions are sources of vital information on child-related issues that can promote adequate nutrition and health. This result is in accordance with the findings of Gebrehaweria and Lemma [75] and Baranwal et al. [81].

The gender of the children showed that male children had a higher likelihood of being anaemic. This is in line with the findings of Assunção et al. [78] but contrary to those of Ewusie et al. [73]. The age of the children was positively associated with being non-anaemic. This is expected due to the higher prevalence of anaemia among infants. Therefore, our findings can be supported by the findings of Assunção et al. [78], Ewusie et al. [73] and Zhao et al. [74] but are contrary to the findings of Gebrehaweria and Lemma [75]. The child’s birth order was found to influence anaemia. Specifically, children who were the fifth-born had a higher probability of being non-anaemic, while second-born children had higher anaemic probability. Our findings are in accordance with those of Moschovis et al. [32] but contrary to those of Yadav et al. [82]. We also found that being the second of multiple births increased the probability of being anaemic. Our finding can be explained from the fact that in some in some instances, the second-born twin may be underweight, thereby promoting growth retardation, stunting, and anaemia [30]. Our finding is in line with those of Asmare and Agmas [83], as well as Seifu and Tesema [30].

We also found that although access to radio did not have a significant effect on prevalence of anaemia, access to newspapers and television had significant influences. This may have reflected the role of media programmes in facilitating awareness and prevention of anaemia among under-5 children. The results are in alignment with those of Baranwal et al. [81] and Anteneh and Van Geertruyden [84], who reported a decrease in the odds of being anaemic with access to media programmes through newspapers, television, and radio.

## 5. Conclusions

The interplay of malaria and other febrile infections in the promotion of anaemia is a subject of significant policy relevance in Africa. In addition, a clear understanding of essential correlates of anaemia is fundamental towards achievement of SDGs, especially the ones that are related to health. This study contributes to the existing body of knowledge on child anaemia and its relationships to malaria infection, fever, and other important correlates in Niger. Our adopted econometric model facilitates the robustness of the estimated parameters through an essential correction of heteroscedasticity. The results have shown a very high incidence of anaemia among under-5 children in Niger, which raises serious questions regarding the efficacy of some on-going programmes for promoting children’s health outcomes by addressing undernutrition and malaria prevalence. The high incidence of malaria necessitates some critical interventions for protecting Nigerien children from mosquito bites and the facilitation of effective healthcare treatment mechanisms for infected children. Of significant note is the fact that other febrile infections which were prevalent among under-5 children had significant impacts on child’s anaemia. Therefore, the existence of a sustainable framework for reducing illnesses among under-5 children will go a long way in addressing anaemia among under-5 children, more so that urban children were better off in all the studied health indicators.

Furthermore, there were some regional differences in anaemia, fever, and malaria prevalence in Niger. This calls for interventions that critically evaluate the vulnerability of each region for some direct marginal reforms. Specifically, children from Tillaberi and Dosso exhibited the highest vulnerability to anaemia. Our results have highlighted the role of information sources in addressing anaemia, with mothers’ access to newspaper and television showing some anaemia prevalence-reducing promise. Therefore, the integration of some essential child and health promoting information focusing on prevention of anaemia in Nigerien newspapers and television hold significant prospects in reducing the current high prevalence of anaemia. Another important finding is the role of maternal fertility, which was captured by children’s birth order, in promoting anaemia. Conventionally, high maternal fertility induces poverty, which will also breed diseases and anaemia among children. Interventions to educate women of reproductive age on the need for family planning may go a very long way in addressing anaemia in Niger. This can be further buttressed by the fact that households’ wealth was an important factor in reducing anaemia among under-5 children. Finally, our results emphasize the role of children’s age and gender in explaining anaemia. This reaffirms the need for an adequate consideration of gender and age in addressing child’s anaemia in Niger with a focus on males and infants.

## Figures and Tables

**Table 1:** Descriptive statistics of selected mothers and children’s variables (n = 4142).

Variables	Mean	Std. Err.	[95% Confidence Interval]	
Mother’s age	28.8752	0.1791	28.5219	29.2285
Agadez	0.0283	0.0036	0.0212	0.0355
Diffa	0.0228	0.0020	0.0189	0.0268
Dosso	0.0798	0.0059	0.0681	0.0914
Maradi	0.2546	0.0186	0.2180	0.2913
Tahoua	0.2021	0.0144	0.1737	0.2304
Tillaberi	0.1342	0.0163	0.1021	0.1664
Zinder	0.2314	0.0144	0.2029	0.2599
Niamey	0.0468	0.0046	0.0377	0.0559
Urban	0.1623	0.0153	0.1322	0.1925
Altitude	354.4323	4.3246	345.9018	362.9628
Primary	0.1498	0.0092	0.1316	0.1679
Secondary	0.0904	0.0073	0.0760	0.1049
Tertiary	0.0064	0.0013	0.0038	0.0091
Household size	8.4648	0.1660	8.1372	8.7923
Male headship	1.0681	0.0082	1.0520	1.0842
Head age	42.5306	0.3562	41.8279	43.2333
Newspaper	0.0151	0.0026	0.0100	0.0203
Radio	0.2569	0.0161	0.2252	0.2885
TV	0.1279	0.0122	0.1038	0.1520
1st birth order	0.5928	0.0082	0.5766	0.6090
2nd birth order	0.3537	0.0069	0.3401	0.3674
3rd birth order	0.0510	0.0041	0.0429	0.0591
4th birth order	0.0021	0.0008	0.0005	0.0037
5th birth order	0.0003	0.0003	−0.0003	0.0010
Not twins	0.9699	0.0044	0.9612	0.9787
1st of multiple	0.0138	0.0021	0.0097	0.0179
2nd of multiple	0.0163	0.0026	0.0112	0.0214
Gender of child	0.5086	0.0093	0.4902	0.5269
Child age	31.3624	0.2606	30.8483	31.8765
Wealth index	2579.2440	5380.3780	−8033.0703	13,192.1900

**Table 2:** Distribution of malaria and fever incidences across regions and sectors in Niger (n = 4142).

Variables	Freq.	Percent	Freq.	Percent
Region	Malaria		Fever	
Agadez	12	2.07	38	2.71
Diffa	22	3.79	97	6.91
Dosso	120	20.69	242	17.25
Maradi	106	18.28	213	15.18
Tahoua	80	13.79	187	13.33
Tillaberi	70	12.07	251	17.89
Zinder	134	23.10	290	20.67
Niamey	36	6.21	85	6.06
Urban/Rural				
Rural	483	83.28	1184	84.39
Urban	97	16.72	219	15.61
Total (% all children)	580	14.00	1403	33.87

**Table 3:** Results of the heteroscedasticity-consistent ordered probit and their marginal values.

Variables	All		Severe Anaemia		Moderate Anaemia		Mild Anaemia		Non-Anaemic	
	Ordered Probit		Marginal Coeff		Marginal Coeff		Marginal Coeff		Marginal Coeff	
	Coeffi	t-stat	Coeffi	t-stat	Coeffi	t-stat	Coeffi	t-stat	Coeffi	t-stat
Malaria	−0.1367	−1.99	0.0110	1.82	0.0436	2.04	−0.0134	−1.8	−0.0411	−2.06
Fever	−0.2396	−4.45	0.0188	3.79	0.0767	4.58	−0.0226	−3.92	−0.0729	−4.60
Mother’s age	0.0049	1.47	−0.0004	−1.47	−0.0016	−1.47	0.0004	1.46	0.0015	1.48
Diffa	0.1344	0.80	−0.0088	−0.89	−0.0446	−0.79	0.0097	1.01	0.0437	0.77
Dosso	−0.0024	−0.01	0.0002	0.01	0.0008	0.01	−0.0002	−0.01	−0.0008	−0.01
Maradi	0.1190	0.90	−0.0083	−0.94	−0.0391	−0.89	0.0096	0.98	0.0378	0.88
Tahoua	0.0872	0.62	−0.0061	−0.65	−0.0286	−0.62	0.0071	0.67	0.0276	0.61
Tillaberi	−0.0433	−0.21	0.0033	0.21	0.0140	0.22	−0.0040	−0.21	−0.0133	−0.22
Zinder	−0.0405	−0.33	0.0030	0.32	0.0131	0.33	−0.0037	−0.32	−0.0125	−0.33
Niamey	0.0738	0.36	−0.0051	−0.38	−0.0243	−0.35	0.0059	0.4	0.0235	0.35
Urban	0.3391	5.44	−0.0204	−5.27	−0.1130	−5.48	0.0200	6.58	0.1134	5.18
Altitude	0.0005	0.97	0.0000	−0.96	−0.0002	−0.98	0.0000	0.96	0.0001	0.98
Primary	0.0750	1.09	−0.0053	−1.13	−0.0246	−1.09	0.0061	1.17	0.0238	1.08
Secondary	−0.0222	−0.26	0.0017	0.25	0.0072	0.26	−0.0020	−0.25	−0.0069	−0.26
Tertiary	−0.0137	−0.06	0.0010	0.06	0.0044	0.06	−0.0012	−0.06	−0.0042	−0.06
Household size	0.0028	0.45	−0.0002	−0.45	−0.0009	−0.45	0.0002	0.45	0.0009	0.45
Male headship	−0.0580	−0.55	0.0043	0.55	0.0189	0.55	−0.0051	−0.55	−0.0181	−0.55
Head age	−0.0011	−0.48	0.0001	0.48	0.0004	0.48	−0.0001	−0.48	−0.0003	−0.48
Newspaper	0.3611	2.50	−0.0193	−3.43	−0.1211	−2.51	0.0156	6.62	0.1248	2.30
Radio	0.0384	0.73	−0.0028	−0.73	−0.0125	−0.73	0.0033	0.75	0.0120	0.73
TV	0.1472	2.03	−0.0098	−2.24	−0.0487	−2.00	0.0110	2.49	0.0476	1.96
2nd Birth order	−0.1189	−2.26	0.0091	2.14	0.0384	2.27	−0.0109	−2.16	−0.0366	−2.28
3rd Birth order	0.0589	0.69	−0.0041	−0.73	−0.0194	−0.69	0.0048	0.75	0.0187	0.68
4th Birth order	−0.3899	−0.58	0.0407	0.43	0.1120	0.71	−0.0490	−0.47	−0.1038	−0.70
5th Birth order	8.5348	46.15	−0.0332	−9.16	−0.4550	−46.31	−0.2724	−28.37	0.7606	89.91
1st of Multiple	−0.1279	−0.80	0.0084	0.9	0.0424	0.79	−0.0094	−1.02	−0.0415	−0.77
2nd of Multiple	−0.4698	−2.29	0.0521	1.63	0.1304	3.02	−0.0613	−1.86	−0.1212	−2.89
Gender of child	−0.0926	−2.18	0.0068	2.21	0.0302	2.16	−0.0081	−2.17	−0.0289	−2.17
Child age	0.0122	6.91	−0.0009	−6.87	−0.0040	−6.58	0.0011	6.44	0.0038	6.78
Wealth index			−6.2 × 10^−8^	−2.53	5.6 × 10^−8^	2.5	1.1 × 10^−7^	2.52	−9.99 × 10^−8^	−2.58
lnsigma										
v191awealth	−4.6 × 10^−7^	−2.54								
/cut1	−1.4143									
/cut2	0.3900									
/cut3	1.1268									
Number of strata	16									
Number of PSUs	206									
Number of obs	4142									
Population size	4,213,981,348									
Design df	190									
F (29,162)	227.57 ***									
Mean VIF	2.39									

***—statistically significant at 1% level.

## Data Availability

Restrictions apply to the availability of these data. Data were obtained from [DHS] and are available [www.dhs.com] with the permission of [DHS].
